# Supplementary material for: Early diagnosis of dengue: Diagnostic utility of the SD BIOLINE Dengue Duo rapid test in Reunion Island
Source: PLoS Negl Trop Dis. 2023 Mar 30;17(3):e0011253. doi: 10.1371/journal.pntd.0011253 (PMC10089357; doi:10.1371/journal.pntd.0011253)
Supplement: S2 Table — Legend: RDT: rapid diagnostic test; NS1 Ag: non-structural 1 antigen; IgM: immunoglobulin M; PLR: positive likelihood ratio; NLR: negative likelihood ratio. (DOCX) [file pntd.0011253.s002.docx]

**S2 Table:** Performance of RDT with duration from illness onset ≤ 5 days vs > 5 days, Reunion, 2019 (N=671)

| **RDT components** | **DIO** | **Sensitivity** | **Specificity** | **PLR** | **NLR** |
| --- | --- | --- | --- | --- | --- |
| **Overall** | **≤ 5 days** | 38 (32-45) | 7 (4-11) | 0.41 (0.35-0.48) | 8.38 (5.38-13.06) |
|  | **> 5 days** | 77 (56-91) | 6 (1-16) | 0.82 (0.65-1.02) | 3.92 (1.07-14.44) |
| **NS1 Ag** | **≤ 5 days** | 11 (7-15) | 75 (68-81) | 0.42 (0.27-0.65) | 1.20 (1.09-1.32) |
|  | **> 5 days** | 26 (10-48) | 86 (71-95) | 1.83 (0.66-5.02) | 0.86 (0.66-1.13) |
| **IgM** | **≤ 5 days** | 25 (19-31) | 27 (21-34) | 0.34 (0.26-0.43) | 2.79 (2.18-3.57) |
|  | **> 5 days** | 61 (39-80) | 21 (10-37) | 0.77 (0.54-1.11) | 1.83 (0.84-3.95) |

RDT : rapid diagnostic test ; NS1 Ag : non-structural 1 antigen ; IgM : immunoglobulin M ; PLR : positive likelihood ratio ; NLR : negative likelihood ratio
