# Supplementary material for: Early diagnosis of dengue: Diagnostic utility of the SD BIOLINE Dengue Duo rapid test in Reunion Island
Source: PLoS Negl Trop Dis. 2023 Mar 30;17(3):e0011253. doi: 10.1371/journal.pntd.0011253 (PMC10089357; doi:10.1371/journal.pntd.0011253)
Supplement: S3 Table — Legend: RDT: rapid diagnostic test; NS1 Ag: non-structural 1 antigen; IgM: immunoglobulin M; PLR: positive likelihood ratio; NLR: negative likelihood ratio. (DOCX) [file pntd.0011253.s003.docx]

**S3 Table:** Performance of NS1 and IgM RDT depending on the duration from illness onset, Reunion, 2019 (N=547)

| **Day** | **N* (%)** | **NS1 RDT sensitivity**  **(%)** | **NS1 RDT specificity**  **(%)** | **IgM RDT sensitivity**  **(%)** | **IgM RDT specificity**  **(%)** |
| --- | --- | --- | --- | --- | --- |
| **0** | 120 (25) | 10 (4-20) | 73 (59-84) | 25 (15-37) | 25 (14-39) |
| **1** | 103 (21) | 8 (3-17) | 76 (60-89) | 15 (8-26) | 39 (24-57) |
| **2** | 71 (15) | 14 (5-29) | 74 (56-87) | 19 (8-35) | 24 (11-41) |
| **3** | 48 (10) | 12 (3-31) | 70 (47-88) | 36 (18-57) | 43 (23-66) |
| **4** | 46 (10) | 17 (5-39) | 87 (66-97) | 39 (20-61) | 13 (3-34) |
| **5** | 28 (6) | 0 (0-34) | 68 (43-87) | 44 (14-79) | 11 (1-33) |
| **6** | 16 (3) | 33 (4-78) | 70 (35-93) | 50 (12-88) | 30 (7-65) |
| **7** | 29 (6) | 27 (6-61) | 94 (73-100) | 72 (39-94) | 0 (0-19) |
| **8** | 5 (1) | 33 (1-91) | 100 (16-100) | 67 (9-99) | 0 (0-84) |
| **>5** | 65 (14) | 26 (10-48) | 86 (71-95) | 61 (39-80) | 21 (10-37) |

* 66 (12%) missing information on the duration from illness onset

RDT : rapid diagnostic test ; NS1 : non-structural 1 ; IgM : immunoglobulin M ; PLR : positive likelihood ratio ; NLR : negative likelihood ratio
