# Supplementary material for: Early diagnosis of dengue: Diagnostic utility of the SD BIOLINE Dengue Duo rapid test in Reunion Island
Source: PLoS Negl Trop Dis. 2023 Mar 30;17(3):e0011253. doi: 10.1371/journal.pntd.0011253 (PMC10089357; doi:10.1371/journal.pntd.0011253)
Supplement: S4 Table — Legend: RDT: rapid diagnostic test; IgG: immunoglobulin G; PLR: positive likelihood ratio; NLR: negative likelihood ratio. (DOCX) [file pntd.0011253.s004.docx]

**S4 Table:** Performance of IgG RDT with duration from illness onset ≤ 5 days vs > 5 days, Reunion, 2019 (N=547)

| **Day** | **Sensitivity (%)** | **Specificity (%)** | **PLR** | **NLR** |
| --- | --- | --- | --- | --- |
| **Global** | 8 (5-12) | 77 (72-82) | 0.34 (0.22-0.55) | 1.20 (1.11-1.29) |
| **≤ 5 days** | 7 (4-11) | 75 (68-81) | 0.28 (0.17-0.48) | 1.24 (1.13-1.35) |
| **> 5 days** | 17 (5-39) | 69 (53-82) | 0.56 (0.21-1.53) | 1.20 (0.91-1.58) |

RDT : rapid diagnostic test ; IgG : immunoglobulin G ; PLR : positive likelihood ratio ; NLR : negative likelihood ratio
