# Supplementary material for: Early diagnosis of dengue: Diagnostic utility of the SD BIOLINE Dengue Duo rapid test in Reunion Island
Source: PLoS Negl Trop Dis. 2023 Mar 30;17(3):e0011253. doi: 10.1371/journal.pntd.0011253 (PMC10089357; doi:10.1371/journal.pntd.0011253)
Supplement: S5 Table — Legend: CI: confidence interval. (DOCX) [file pntd.0011253.s005.docx]

**S5 Table:** Post-test probabilities and their 95% confidence interval for the RDT according to pre-test probability of dengue, Reunion, 2019 (N=671)

| **Pre-test probability of dengue** | **5%** | **10%** | **20%** | **30%** |
| --- | --- | --- | --- | --- |
| **Post-test probability of dengue for positive test component, % (95%CI)** | 3 (2-4) | 5 (4-7) | 11 (9-13) | 18 (15-20) |
| **Post-test probability of dengue for negative test component, % (95%CI)** | 17 (13-23) | 30 (25-36) | 49 (43-56) | 63 (57-68) |

CI : confidence interval
