# Supplementary material for: Early diagnosis of dengue: Diagnostic utility of the SD BIOLINE Dengue Duo rapid test in Reunion Island
Source: PLoS Negl Trop Dis. 2023 Mar 30;17(3):e0011253. doi: 10.1371/journal.pntd.0011253 (PMC10089357; doi:10.1371/journal.pntd.0011253)
Supplement: Attachment — Submitted filename: Response to reviewers.docx [file pntd.0011253.s007.docx]

Dear Drs Puneet Bhatt, Elvina Viennet and Reviewers,

Please find attached our revised manuscript (PNTD-D-22-01198), entitled ‘Early diagnosis of dengue: diagnostic utility of the SD BIOLINE Dengue Duo rapid test in Reunion Island,’ by Olivier Maillard; Jeanne Bélot; Thibault Adenis; Olivier Rollot; Antoine Adenis; Bertrand Guihard; Patrick Gérardin; Antoine Bertolotti, which we are resubmitting for publication as an Original Article in *PLOS Neglected Tropical Diseases.*

We want to sincerely thank all of you for your useful remarks that have helped us improve our paper and clarify its message. We took into consideration all your comments in revising our manuscript.

Our responses to the comments are detailed below. Comments are in blue, responses are in black and recommended changes are in red in the text and in the manuscript.

**REVIEWER 1**

**Comments:**

1. In the Flow chart Fig 1; Information regarding break up of RT-PCR positive in NS1Ag positive and IgM positive samples would be useful for the reason being IgM positive samples by RDT which were RT-PCR negative (being used as Reference standard) would have resulted inputs regarding any cross reactive antibodies to other closely related Flaviviruses such as West Nile or Japanese encephalitis viruses. This could have been resolved by more specific µ-capture IgM ELISA test in such samples.

2. Whether RT-PCR inhibitors were taken care of in the samples which were found negative by RT-PCR but were found positive by RDT for Dengue antibodies?

3. As seen from S3 table “Performance of NS1 and IgM RDT….”, column “4” on IgM RDT; sensitivity percentage on Day 1 being highest (65%) as compared to other subsequent days including more than 5 days (61%) where as my understanding is that Dengue IgM antibody rise in blood is after 3-5 days and can remain detectable till 90 days.

4. Similarly, NS1 Ag detection by RDT beyond 05 days is indicative of lasting viremia and could have been interesting to see its co-relation with viral load copy number by real time RT-PCR. It is interesting finding which could suggest changing viral dynamics. (For Reference: J Clin. Diagn.Res 2016 Apr; 10 (4):1-4) where authors have found NS1Ag positivity maximum on day 2-5 post onset of illness.

**Responses:**

We wish to thank the reviewer for his very helpful comments

1. In the Flow chart Fig 1; Information regarding break up of RT-PCR positive in NS1Ag positive and IgM positive samples would be useful for the reason being IgM positive samples by RDT which were RT-PCR negative (being used as Reference standard) would have resulted inputs regarding any cross reactive antibodies to other closely related Flaviviruses such as West Nile or Japanese encephalitis viruses. This could have been resolved by more specific µ-capture IgM ELISA test in such samples.

We tried to follow the prototypical STARD 2015 flow diagram but we agree with the reviewer that some relevant information was lacking, and therefore we have modified Figure 1 accordingly (page 8, line 163). New boxes were added to the flowchart to document the frequency of positive components of the RDT among positive RT-PCR. We also added a legend to clarify abbreviations of the figure (page 9, lines 165-166): “RDT: rapid diagnostic test; RT-PCR: reverse transcriptase polymerase chain reaction; NS1 Ag: non-structural 1 antigen; IgM: immunoglobulin M; IgG: immunoglobulin G”.

No circulation of other flaviviruses has been reported on Reunion Island over the study period, and the Tropical Fever Core multiplex real time RT-PCR from Fast Track Diagnostics, which was used as reference standard in our study, could test for the following pathogens: dengue, chikungunya and West Nile viruses, *Leptospira spp.*, *Rickettsia spp.* and *Salmonella spp.*, and *Plasmodium spp.* Serology was done, and helpful in cases of doubt. On this basis, no co-infections with these pathogens were observed in the confirmed or suspected dengue patients enrolled in our study.

But most IgM ELISA were made only once, and not necessarily at the time of RDT, so a diagnosis of ongoing dengue could not be assessed and IgM ELISA could not be taken as a reference test. This is one reason why we focused on the diagnostic utility of RDT in point-of-care patient management, with RT-PCR only as reference, as the study was conducted on patients and not on a convenient sample collection, which would have allowed more accurate analyses. We modified the discussion to better justify this limitation (page 15, lines 275-278): “Furthermore, the reference diagnostic test was only RT-PCR because most patients consulted within the first five days of illness, thus a few had ~~with~~ a serology done at the time of RDT, with ~~had~~ often only one IgM ELISA result in their medical file, and a positive result might represent either an active or a previous, recent infection.”

2. Whether RT-PCR inhibitors were taken care of in the samples which were found negative by RT-PCR but were found positive by RDT for Dengue antibodies?

Most of the time, in the case of PCR inhibitors which could be detected by negative internal controls, results are rendered non interpretable or doubtful but not negative and a new sample is often required or a second RNA extraction.

We have added the following sentence in the method section (page 6, lines 114-116): “In the case of PCR inhibitors, which could be detected by negative internal controls, results were rendered non interpretable or doubtful but not negative, and a new sample was required or a second RNA extraction.”

3. As seen from S3 table “Performance of NS1 and IgM RDT….”, column “4” on IgM RDT; sensitivity percentage on Day 1 being highest (65%) as compared to other subsequent days including more than 5 days (61%) where as my understanding is that Dengue IgM antibody rise in blood is after 3-5 days and can remain detectable till 90 days.

There was a mistake in S3 Table and obviously sensitivity of RDT IgM at Day 0 is much weaker at 25%. S3 Table was modified accordingly (supporting information, page 4, line 9): “25 (15-37)”. We also add this paragraph in the discussion (page 14, lines 256-264): “When focusing on the 89 positive IgM RDT at Day 0 and Day 1 of the onset of illness, only 27 (30%) were RT-PCR positive and 67 (75%) had a serology test but only five (6%) were positive in IgM antibodies. As they were also positive in IgG antibodies, these five patients seemed to be in the convalescent phase of a previous dengue fever. However three also had a positive RT-PCR that could be explained by a secondary dengue, but serum neutralization was not routinely made in 2019 because Reunionese people were presumed to be naïve to dengue. Even if some patients may have mistaken the date of onset of symptoms, these results also illustrate the bad performance of IgM RDT component compared to IgM ELISA, due to a high rate of false positives at Day 0 and Day 1.”. If considering IgM ELISA as reference for IgM RDT, sensitivity and specificity of IgM RDT increased to 44%, IC95%(27-62) and 42%, IC95%(37-48) respectively. However, some IgM ELISA were prescribed after negative RT-PCR, thus not in the same timeframe as the RDT to be compared with, and neither IgM RDT nor a single IgM ELISA are useful for an early diagnosis of dengue (because 87% of patients consulted within the first 5 days after declared illness onset). For a better comprehension, we have added a column with the number and proportion of patients at each day of presentation after illness onset (supporting information, page 4, line 9).

4. Similarly, NS1 Ag detection by RDT beyond 05 days is indicative of lasting viremia and could have been interesting to see its co-relation with viral load copy number by real time RT-PCR. It is interesting finding which could suggest changing viral dynamics. (For Reference: J Clin. Diagn.Res 2016 Apr; 10 (4):1-4) where authors have found NS1Ag positivity maximum on day 2-5 post onset of illness.

The Tropical Fever Core multiplex real time RT-PCR, which was routinely done on-site while patient management, was not a quantitative PCR, so viral loads could not have been estimated and the comparison with NS1 Ag RDT positivity was not possible. We have added this passage in the method section (page 5-6, lines 109-114): “The reference laboratory test for dengue diagnosis confirmation in the study was the Tropical Fever Core multiplex RT-PCR (Fast Track Diagnostics®), which was a conventional, two step, real-time RT-PCR used for on-site diagnosis at the time of the epidemic. This kit was chosen as routine, as it could detect dengue (without differentiation between serotypes), chikungunya and West Nile viruses, *Leptospira spp.*, *Rickettsia spp.* and *Salmonella spp.*, and *Plasmodium spp.,* which are pathogens that could circulate in the South West Indian Ocean region.” Indeed, a purpose of further study could be to monitor NS1 Ag RDT positivity in relation to viral load by using a quantitative RT-PCR.

**REVIEWER 2**

**Comments:**

- The authors made only patients record review only in this study. Is it possible to say retrospective study?

What is the main topics of this manuscript. According to the title, we understood that the usefulness of RDT kits for diagnosis of dengue infection by comparing gold standard tests. But in this study, the authors did not use the full set of gold standard tests for confirmation of IgM Ab. The authors described that gold standard tests used RT-PCR. It is not full set because the authors also used IgM Ab for diagnosis of acute DENV infection. The authors should also test one gold standard IgM Ab tests which is validated for no cross reactivity with other flaviviruses. This is the biggest weak point of this study and the test performance is decreased due to not include IgM Ab at gold standard.

According to the flowchart Fig-1, it is very difficult to interpret for the readers. The authors described step by step but only 325 samples done for reference tests. So the sample size only 325. For calculation of the sensitivity, specificity, all sample must do reference tests to check your RDT test kit is correct or not? The author need to analyse only 325 samples and the authors must do gold standard IgM Ab ELISA test for publication to meet the standard at the reputed international journal.

- At Table-1. The authors described the demographic data and clinical features of study population. Concerning gender, we noted Male” population but cannot find for Female anywhere. Please revise it.

In table 1, RDT was tested on 671 patients. But according to case categories, total number is only 547. What are the discrepancies? And please check it.

As you mentioned that the sensitives were slightly better beyond 5th day, if possible please show the comparison of sensitivities based on day of illness

- The authors described that the standard test as RT-PCR. Please kindly mention that is IT either quantitative or conventional Reverse transcription Polymerase Chain Reaction (RT-PCR)? Moreover, the author should described that it is either one step or two steps? Please also describe the vaidity of this PCR system for cross reactivity of the primers and other flaviviruses and within four serotypes of DENV infection.

In this study, the authors made RT-PCR test Is there any information about the serotype of DENV circulating at this DENV season. It will be interesting to the scientific community and now it is no more new information for the peer group scientific community.

- the RT-PCR test carried out in this study was Tropical Fever Core multiplex RT-PCR (Fast Track Diagnostics®). This is a Multiplex RT-PCR having multiple targets apart from Dengue.

a.	It is not understood as to why a Multiplex RT-PCR kit was used while studying a single virus i.e. Dengue.

**Responses:**

We wish to thank the reviewer for his very helpful comments

- The authors made only patients record review only in this study. Is it possible to say retrospective study?

What is the main topics of this manuscript. According to the title, we understood that the usefulness of RDT kits for diagnosis of dengue infection by comparing gold standard tests. But in this study, the authors did not use the full set of gold standard tests for confirmation of IgM Ab. The authors described that gold standard tests used RT-PCR. It is not full set because the authors also used IgM Ab for diagnosis of acute DENV infection. The authors should also test one gold standard IgM Ab tests which is validated for no cross reactivity with other flaviviruses. This is the biggest weak point of this study and the test performance is decreased due to not include IgM Ab at gold standard.

According to the flowchart Fig-1, it is very difficult to interpret for the readers. The authors described step by step but only 325 samples done for reference tests. So the sample size only 325. For calculation of the sensitivity, specificity, all sample must do reference tests to check your RDT test kit is correct or not? The author need to analyse only 325 samples and the authors must do gold standard IgM Ab ELISA test for publication to meet the standard at the reputed international journal.

It is a retrospective study from a retrospective data collection, as mentioned in the method section (page 5, line 88). Patients’ record review has been conducted on the whole initial sample for studying dengue fever in Reunion.

The aim of this work is to evaluate the performance, and therefore utility, of the RDT for an early point-of-care diagnosis of dengue, as 87% of patients consulted within the first 5 days after declared illness onset. We have added the number and proportion of patients who consulted within the first 5 days and beyond in Table 1 (page 10), and in the discussion (page 13, line 228), and improved the discussion (page 15, lines 275-278): “Furthermore, the reference diagnostic test was only RT-PCR because most patients consulted within the first five days of illness, thus a few had a serology done at the time of RDT, with often only one IgM ELISA result in their medical file, and a positive result might represent either an active or a previous, recent infection.”

Dengue virus-specific IgM and neutralizing antibodies typically develop towards the end of the first week of illness. IgM levels are variable, but generally are positive starting 4-5 days after the onset of symptoms. Moreover, most IgM-ELISA were made only once, so a diagnosis of ongoing dengue could not be assessed. That is the reason why we focused on the performance and diagnostic utility of RDT in comparison with RT-PCR only as reference standard, and in point-of-care patient management, as the study was conducted on patients and not on a convenient sample collection, which would have allowed more accurate analyses. Therefore all RDT were not followed by RT-PCR or IgM ELISA. It resulted in 671 patients with RDT and PCR outcomes of whom 325 with positive RT-PCR.

- At Table-1. The authors described the demographic data and clinical features of study population. Concerning gender, we noted Male” population but cannot find for Female anywhere. Please revise it.

In table 1, RDT was tested on 671 patients. But according to case categories, total number is only 547. What are the discrepancies? And please check it.

As you mentioned that the sensitives were slightly better beyond 5th day, if possible please show the comparison of sensitivities based on day of illness

We agree with the reviewer. Female gender needed to be presented and information was added in Table 1 (page 9, line 179).

The difference in the number of patients enrolled in overall analyses (n=671) and in analyses by component of the RDT (n=547) is related to the lack of information on component results in some patient files. It was already mentioned in the discussion as a selection bias (page 14, lines 270-272) but we have added this limit in the method section (page 7, line 147-148): “...if information on component results was available in patients’ files”. Having excluded these results would have biased analyses and overall RDT performance estimation.

Sensitivities were globally better but there were only few patients who consulted beyond 5 days of illness, so reporting performance for each day beyond resulted in large 95% confidence intervals. However, as requested, we modified the S3 Table accordingly till Day 8 (supporting information, page 4, line 9), and added a column with the number and proportion of patients at each day of presentation after illness onset. Information on missing data in the duration of illness onset is now mentioned below the table (line 10): “66 (12%) missing information on the duration from illness onset.”

**S3 Table:** Performance of NS1 and IgM RDT depending on the duration from illness onset, Reunion, 2019 (N=547)

| **Day** | **N* (%)** | **NS1 RDT sensitivity**  **(%)** | **NS1 RDT specificity**  **(%)** | **IgM RDT sensitivity**  **(%)** | **IgM RDT specificity**  **(%)** |
| --- | --- | --- | --- | --- | --- |
| **0** | 120 (25) | 10 (4-20) | 73 (59-84) | 25 (15-37) | 25 (14-39) |
| **1** | 103 (21) | 8 (3-17) | 76 (60-89) | 15 (8-26) | 39 (24-57) |
| **2** | 71 (15) | 14 (5-29) | 74 (56-87) | 19 (8-35) | 24 (11-41) |
| **3** | 48 (10) | 12 (3-31) | 70 (47-88) | 36 (18-57) | 43 (23-66) |
| **4** | 46 (10) | 17 (5-39) | 87 (66-97) | 39 (20-61) | 13 (3-34) |
| **5** | 28 (6) | 0 (0-34) | 68 (43-87) | 44 (14-79) | 11 (1-33) |
| **6** | 16 (3) | 33 (4-78) | 70 (35-93) | 50 (12-88) | 30 (7-65) |
| **7** | 29 (6) | 27 (6-61) | 94 (73-100) | 72 (39-94) | 0 (0-19) |
| **8** | 5 (1) | 33 (1-91) | 100 (16-100) | 67 (9-99) | 0 (0-84) |
| **>5** | 65 (14) | 26 (10-48) | 86 (71-95) | 61 (39-80) | 21 (10-37) |

* 66 (12%) missing information on the duration from illness onset

RDT : rapid diagnostic test ; NS1 : non-structural 1 ; IgM : immunoglobulin M ; PLR : positive likelihood ratio ; NLR : negative likelihood ratio

- The authors described that the standard test as RT-PCR. Please kindly mention that is IT either quantitative or conventional Reverse transcription Polymerase Chain Reaction (RT-PCR)? Moreover, the author should described that it is either one step or two steps? Please also describe the vaidity of this PCR system for cross reactivity of the primers and other flaviviruses and within four serotypes of DENV infection.

In this study, the authors made RT-PCR test Is there any information about the serotype of DENV circulating at this DENV season. It will be interesting to the scientific community and now it is no more new information for the peer group scientific community.

Indeed it was a conventional, two step, multiplex, real-time RT-PCR. We modified the manuscript in the method section (page 5-6, line 108-114): “The reference laboratory test for dengue diagnosis confirmation in the study was the Tropical Fever Core multiplex RT-PCR (Fast Track Diagnostics®), which was a conventional, two step, real-time RT-PCR used for on-site diagnosis at the time of the epidemic. This kit was chosen as routine, as it could detect dengue (without differentiation between serotypes), chikungunya and West Nile viruses, *Leptospira spp.*, *Rickettsia spp.* and *Salmonella spp.*, and *Plasmodium spp.*, which are pathogens that could circulate in the South West Indian Ocean region”. We also referred to manufacturer protocols at this time because the kit is now for research use only since Siemens has purchased FTD and added the following sentence (page 6, line 116): “All procedures were performed according to the manufacturer’s protocols. Serology was not done as routine, but when there was a positive IgM RDT or a strong suspicion of dengue fever and a negative RT-PCR result.”. This is true for any fever which motivated us to prescribe the multiplex RT-PCR, but we focused on dengue in the manuscript.

The DENV-2 serotype has been mostly identified over the study period, as mentioned in the discussion (page 14, lines 266-268), but it was based on a sample of patients because the RT-PCR kit did not allow dengue differentiation and serotype was not determined routinely.

- the RT-PCR test carried out in this study was Tropical Fever Core multiplex RT-PCR (Fast Track Diagnostics®). This is a Multiplex RT-PCR having multiple targets apart from Dengue.

a.	It is not understood as to why a Multiplex RT-PCR kit was used while studying a single virus i.e. Dengue.

The RT-PCR kit was used for on-site routine diagnosis and not for research. Reunion is a tropical island, so this multiplex RT-PCR is indicated in cases of fever, even during a dengue outbreak. This also allows us to discard other circulating pathogens, which can occur because Reunion is connected to several places in the South West Indian Ocean: Madagascar, Mauritius, Seychelles, Comoros, South Africa, Mozambique, Kenya, plus many more destinations via Mauritius, an international travel hub. We then added this information (page 6, lines 111-114): “This kit was chosen as routine, as it could detect dengue (without differentiation between serotypes), chikungunya and West Nile viruses, *Leptospira spp.*, *Rickettsia spp.* and *Salmonella spp.*, and *Plasmodium spp.*, which are pathogens that could circulate in the South West Indian Ocean region.”

**REVIEWER 3**

**Comments:**

- Sample size calculation not mentioned

**Response:**

This study is based on a retrospective cohort composed of all the patients with a suspected diagnosis of dengue at initial presentation in emergency rooms of the University Hospital during the 2019 outbreak in Reunion. Thus, the sample size derived from the availability of results of the RDT used at point-of-care during this epidemic, and the RT-PCR used for on-site routine diagnosis over this period.

In this conditions (with a sample dengue prevalence of 0.46), a sample size of 547 patients produces a two-sided 95% confidence interval with a width ranging from 0.078 to 0.127 for component sensitivities from 0.1 to 0.9, and from 0.072 to 0.117 for a component specificities from 0.1 to 0.9. Therefore the sample size is adequate to estimate accurate sensitivities and specificities. A paragraph was added in the discussion (page 14, lines 272-274): “However, a sample size of 547 patients is adequate to estimate accurate performances as it produces a two-sided 95% confidence interval with a width ranging from 0.07 to 0.13 for component sensitivities or specificities from 0.10 to 0.90.”.

We hope you will find our revised version acceptable for publication in *PLOS Neglected Tropical Diseases* and look forward to hearing from you.

Yours sincerely,

Dr Olivier MAILLARD, PharmD, MSPH

Dr Antoine BERTOLOTTI, MD, PhD
