# Supplementary material for: Early diagnosis of dengue: Diagnostic utility of the SD BIOLINE Dengue Duo rapid test in Reunion Island
Source: PLoS Negl Trop Dis. 2023 Mar 30;17(3):e0011253. doi: 10.1371/journal.pntd.0011253 (PMC10089357; doi:10.1371/journal.pntd.0011253)
Supplement: Attachment — Submitted filename: Early diagnosis of dengue.docx [file pntd.0011253.s008.docx]

Early diagnosis of dengue: diagnostic utility of the SD 2 BIOLINE Dengue Duo rapid test in Reunion Island by Olivier Maillard et al.

1. Line number 65: Global burden or burden in Reunion island? The mortality rate also needs to be mentioned.
2. Line number 70: The meaning is unclear “over these years”. Authors meant to say from 2018?
3. Line number 84: SD Bioline Dengue Duo RDT- Manufacture’s details are not given ( country of origin, catalogue number etc.
4. Line number 80-84 : The rationale of the study could have been defined a little more.
5. Line number 88 : Details need to be added about the EPIDENGUE database
6. Line number 92: Why was only the age group of 18 years and above considered? What are the inclusion criteria, and exclusion criteria? Is the RDT performed only for selected patients?
7. Line 88-100: the sample size is not included
8. Line number 100: real-time RT-PCR or conventional RT-PCR
9. Line number 101: May be changed from Diagnostic tests to Diagnostic test/ RDT under evaluation
10. Line number 102-108: RDT is validated only for acute infection? In secondary dengue also, IgG can be present. Authors may relook into the statement.

Test validity also not mentioned? i.e control line band

11. Line number 114-118: The meaning is not clear. Rewrite the sentence for better clarity.
12. Line number 117-118: Sentence is incomplete, please look into the sentence.
13. Line number 101-118: No mention of reference standard used against the kit under evaluation (Refer Banoo S, Bell D, Bossuyt P, et al. Evaluation of diagnostic tests for infectious diseases: general principles. Nat Rev Microbiol. 2006;4(9 Suppl):S21-S31. doi:10.1038/nrmicro1523)
14. Line number 101, 119- what is the importance of diagnostic tests used and final diagnosis? The evaluation was done retrospectively. So here you need what are the criteria to be enrolled as positive and negative. Sample size needs to be calculated for positive and negative samples based on the prevalence, expected sensitivity, specificity, PPV, and NPV.
15. Line number 155, the sample size for RDT was 946 or 671?
16. Line number 155-162; better to provide data in a tabular form clearly indicating the details of RDT, RT-PCR and ELISA to avoid confusion
17. Line number 168-178: Is clinical and demographic data relevant here?
18. Line number 179: The research article title says diagnostic assay evaluation. Then Table 1 is not relevant
19. Line number 194: Table 2: How did the authors compare the performance valuation? There is no information about reference standards.
20. Line number 199-204: How is the sensitivity correlated based on days of onset? In the majority of cases, NS1 detection is best in the first 5 days of illness. Authors, please check the disease kinetics of dengue virus infection. NS1 RDT shall either compare with NS1 ELISA or PCR and IgM RDT shall compare with IgM ELISA (Reference standard)
21. Line number 221: The discussion section needs substantial improvement.

Available RDT for dengue detection and comparison with other findings (sensitivity, specificity- etc), Future implications etc.

22. Line number 279: Conclusion: The authors need more information to conclude the study. Based on current findings, it is hard to comment on the sensitivity and specificity of the RDT.
