# Supplementary material for: Early diagnosis of dengue: Diagnostic utility of the SD BIOLINE Dengue Duo rapid test in Reunion Island
Source: PLoS Negl Trop Dis. 2023 Mar 30;17(3):e0011253. doi: 10.1371/journal.pntd.0011253 (PMC10089357; doi:10.1371/journal.pntd.0011253)
Supplement: Attachment — Submitted filename: Response to reviewers.docx [file pntd.0011253.s009.docx]

Dear Dr Elvina Viennet and Reviewers,

Please find attached our revised manuscript (PNTD-D-22-01198), entitled ‘Early diagnosis of dengue: diagnostic utility of the SD BIOLINE Dengue Duo rapid test in Reunion Island,’ by Olivier Maillard; Jeanne Bélot; Thibault Adenis; Olivier Rollot; Antoine Adenis; Bertrand Guihard; Patrick Gérardin; Antoine Bertolotti, which we are resubmitting for publication as an Original Article in *PLOS Neglected Tropical Diseases.*

We want to sincerely thank all of you for your useful remarks that have helped us improve our paper and clarify its message. We took into consideration all your comments in revising our manuscript.

Our responses to the comments are detailed below. Comments are in blue, responses are in black and recommended changes are in red in the text and in the manuscript.

Comment:

1.	Line number 65: Global burden or burden in Reunion island? The mortality rate also needs to be mentioned.

Response: We have added information in the text: “The massive increase of dengue incidence, with 400 million estimated annual cases worldwide combined with the geographical extension of its vector, the Aedes mosquito, makes dengue a major public health concern”

Comment:

2.	Line number 70: The meaning is unclear “over these years”. Authors meant to say from 2018?

Response: To avoid any misunderstanding, we have removed these words: “Dengue outbreaks have been growing in Reunion Island since 2018, with an increasing number of confirmed cases and related deaths reported ~~over these years”~~

Comment:

3.	Line number 84: SD Bioline Dengue Duo RDT- Manufacture’s details are not given ( country of origin, catalogue number etc.

Response: We have modified as mostly reported in other articles (thus without the catalog number): “The aim of this retrospective study was to assess the performance of the SD Bioline Dengue Duo RDT (Standard Diagnostics, South Korea) in Reunion Island during the 2019 epidemic.” And further (line 120): “The reference laboratory test for dengue diagnosis confirmation in the study was the Tropical Fever Core multiplex RT-PCR (Fast Track Diagnostics, Luxembourg), which was a conventional, two step, real-time RT-PCR used for on-site diagnosis at the time of the epidemic.”

Comment:

4.	Line number 80-84 : The rationale of the study could have been defined a little more.

Response: After several readings we do not find what to add as rationale but maybe as an objective to the introduction: “and whether physicians could make a diagnosis on the basis of its results in this setting”

Comment:

5.	Line number 88 : Details need to be added about the EPIDENGUE database

Response: The EPIDENGUE database was described and the registration number in the French health data hub was mentioned but we have added the following information to clarify its main goal: “The EPIDENGUE database was constituted to study dengue fever in Reunionese patients during 2019 epidemic while a prospective study was launched in the same time but with limited recruitment as emergency departments were overwhelmed during the outbreak.”

Comment:

6.	Line number 92: Why was only the age group of 18 years and above considered? What are the inclusion criteria, and exclusion criteria? Is the RDT performed only for selected patients?

Response: Only 10 children had both RDT and RT-PCR, so we decided not to include them. This may be related to the clinical diagnosis of dengue, which is more difficult to establish in children whose features are often mild and varied. We have modified as follow:” A retrospective study of diagnostic accuracy of SD Bioline dengue duo RDT was conducted in patients over 18 years of age because only few children had both RDT and RT-PCR”

Comment:

7.	Line 88-100: the sample size is not included

Response: The study is based on a retrospective cohort composed of all cases with a suspected diagnosis of dengue in emergency rooms of the University Hospital during the 2019 epidemic in Reunion.

A paragraph has already been added in the discussion but as requested we have also added the following information in the method section: “Sample size was determined based on the availability of the point-of-care RDT and RT-PCR results used for routine on-site diagnosis during the study period.

Under these conditions, a sample of 547 patients yields a two-sided 95% confidence interval with a width ranging from 0.078 to 0,127 for component sensitivities of 0.1 to 0.9, and ranging from 0.072 to 0.117 for component specificities of 0.1 to 0.9. Sample size calculation was performed using PASS software (PASS 2020, NCSS, LLC, Kaysville, Utah, USA).”

We have therefore decided to delete the same information that was added in the discussion of the previous revision: “~~However, a sample size of 547 patients is adequate to estimate accurate performances as it produces a two-sided 95% confidence interval, with a width ranging from 0.07 to 0.13 for component sensitivities or specificities from 0.10 to 0.90.~~

Comment:

8.	Line number 100: real-time RT-PCR or conventional RT-PCR

Response: We changed the text to "Patients suspected of having dengue fever underwent point-of-care testing by blood sampling on admission. All had an RDT evaluated by a primary care physician, and a few also had a reference test, i.e. real-time RT-PCR during the acute phase…"

Comment:

9.	Line number 101: May be changed from Diagnostic tests to Diagnostic test/ RDT under evaluation

Response: We have modified accordingly: “The ~~evaluated RDT~~ diagnostic test under study was the SD Bioline Dengue Duo (Standard Diagnostics, South Korea). This is a rapid immunochromatographic one-step assay to detect simultaneously the NS1 antigen and IgM and IgG DENV antibodies.”

Comment:

10.	Line number 102-108: RDT is validated only for acute infection? In secondary dengue also, IgG can be present. Authors may relook into the statement.

Test validity also not mentioned? i.e control line band

Response: It is written in the manuscript: “IgG antibody results were not considered because they are not used to validate the diagnosis of acute infection in current practice”. That does not mean that the RDT is only validated for acute infections ! And it is clear that IgG can be present with IgM antibodies or NS1 antigen in secondary dengue. But in these cases, the IgM or NS1 results were taken into account provided that a RT-PCR was performed at the same time as a reference standard. Furthermore, it is already mentioned: “The presence of colored lines in both results windows (control and patient) indicates a positive result. A faint line in NS1 or IgM antibodies was considered positive, in accordance with the manufacturer’s instructions.”

Comment:

11.	Line number 114-118: The meaning is not clear. Rewrite the sentence for better clarity.

Response: We would like to apologize to the reviewer but after several readings we do not see what to clarify.

Comment:

12.	Line number 117-118: Sentence is incomplete, please look into the sentence.

Response: We have modified as follow: “Serology was not done routinely, but when there was a positive IgM RDT or a strong suspicion of dengue fever and a negative RT-PCR result.”

Comment:

13.	Line number 101-118: No mention of reference standard used against the kit under evaluation (Refer Banoo S, Bell D, Bossuyt P, et al. Evaluation of diagnostic tests for infectious diseases: general principles. Nat Rev Microbiol. 2006;4(9 Suppl):S21-S31. doi:10.1038/nrmicro1523)

Response: The reference standard was the Tropical Fever Core multiplex RT-PCR (FTD, Luxembourg) as mentioned line 119: “The reference laboratory test for dengue diagnosis confirmation in the study was the Tropical Fever Core multiplex RT-PCR (Fast Track Diagnostics, Luxembourg), which was a conventional, two step, real-time RT-PCR used for on-site diagnosis at the time of the epidemic.”

Comment:

14.	Line number 101, 119- what is the importance of diagnostic tests used and final diagnosis? The evaluation was done retrospectively. So here you need what are the criteria to be enrolled as positive and negative. Sample size needs to be calculated for positive and negative samples based on the prevalence, expected sensitivity, specificity, PPV, and NPV.

Response:

This study is based on a retrospective cohort composed of all cases with a suspected diagnosis of dengue in emergency rooms of the University Hospital during the 2019 epidemic in Reunion Island. The performance and utility of the RDT under study should take into account the final diagnosis as recommended by the STARD guidelines.

A paragraph has already been added in the discussion but as requested we have also added the following information in the method section: “Sample size was determined based on the availability of the point-of-care RDT and RT-PCR results used for routine on-site diagnosis during the study period.

Under these conditions, a sample of 547 patients yields a two-sided 95% confidence interval with a width ranging from 0.078 to 0,127 for component sensitivities of 0.1 to 0.9, and ranging from 0.072 to 0.117 for component specificities of 0.1 to 0.9. Sample size calculation was performed using PASS software (PASS 2020, NCSS, LLC, Kaysville, Utah, USA).”

It is unusual to report a sample size calculation based on predictive values as they derived from the sensitivity, specificity and prevalence of the disease and of the positive or negative test in the sample, which are results of the study and not assumptions to present in the method section. It can also be evidenced by the fact that this method is not available in our sample size calculation software PASS 2020 (NCSS, LLC, Kaysville, Utah, USA) which is one of the most widely used in the research field in the world.

Comment:

15.	Line number 155, the sample size for RDT was 946 or 671?

Response: In the retrospective EPIDENGUE cohort, 946 patients underwent the RDT under study but only 671 also underwent RT-PCR as a reference standard. Therefore 671 RDT results could be evaluated as shown in the flowchart (figure 1).

Comment:

16.	Line number 155-162; better to provide data in a tabular form clearly indicating the details of RDT, RT-PCR and ELISA to avoid confusion

Response: A table might be more confusing than a flowchart that is required by the STARD guidelines (S1 table). To comply with the guidelines, we decided to keep the flowchart.

Comment:

17.	Line number 168-178: Is clinical and demographic data relevant here?

Response: Because the performance of such RDTs is very depending on the setting and the study is based on patients rather than on a sample collection, we believe that the presentation of patients’ characteristics is relevant in the manuscript and is also recommended by the STARD guidelines (S1 table).

Comment:

18.	Line number 179: The research article title says diagnostic assay evaluation. Then Table 1 is not relevant

Response: Because the performance of such RDTs is very depending on the setting and the study is based on patients rather than on a sample collection, we believe that table 1 is relevant in the manuscript and is also recommended by the STARD guidelines (S1 table).

Comment:

19.	Line number 194: Table 2: How did the authors compare the performance valuation? There is no information about reference standards.

Response: The reference standard was the Tropical Fever Core multiplex RT-PCR (FTD, Luxembourg) as mentioned line 119: “The reference laboratory test for dengue diagnosis confirmation in the study was the Tropical Fever Core multiplex RT-PCR (Fast Track Diagnostics, Luxembourg), which was a conventional, two step, real-time RT-PCR used for on-site diagnosis at the time of the epidemic.”

Comment:

20.	Line number 199-204: How is the sensitivity correlated based on days of onset? In the majority of cases, NS1 detection is best in the first 5 days of illness. Authors, please check the disease kinetics of dengue virus infection. NS1 RDT shall either compare with NS1 ELISA or PCR and IgM RDT shall compare with IgM ELISA (Reference standard)

Response: These are results in our sample that are discussed in more details in the appropriate section. Only 12% of patients consulted after 5 days from the onset of the disease, so the 95% confidence intervals (CI) are larger than in the first five days (S3 table). Therefore, depending on the sampling conditions, it could also be the other way around because the 95%CI of sensitivity is 7-15 for NS1 when ≤ 5 days of illness and 10-48 after 5 days, and the 95%CI of specificity is 68-81 when ≤ 5 days of illness and 71-95 after 5 days. But it is true that in general, NS1 antigen is an alternative to RT-PCR in the first five days of illness. Serology is then recommended.

The aim of the study was to evaluate the performance and therefore the utility of the RDT for early diagnosis at point-of-care as 88% of patients consulted within the first 5 days after illness onset which is declarative and corresponds to an approximate date.

In addition, we agree with the reviewer that the NS1 RDT should have been compared with the NS1 ELISA but this was not done routinely in 2019, and most IgM ELISA were performed only once and not necessarily at the time of RDT so an ongoing diagnosis of dengue could not be evaluated and neither IgM ELISA nor NS1 ELISA could not be considered reference tests in our study.

Comment:

21.	Line number 221: The discussion section needs substantial improvement.

Available RDT for dengue detection and comparison with other findings (sensitivity, specificity- etc), Future implications etc.

Response: Submitted as a short communication, we have attempted to synthesize the revised manuscript into 2,500 words. Moreover, we have already addressed some comparisons with other results obtained with the same or other RDTs as well as future implications although the most important underlying but not written one was to change the RDT for the 2020 epidemic. Physicians noted the poor performance of the RDT in our setting while using it at point-of-care; our study just corroborated their impression.

Comment:

22.	Line number 279: Conclusion: The authors need more information to conclude the study. Based on current findings, it is hard to comment on the sensitivity and specificity of the RDT.

Response: Since the aim is to evaluate the RDT as a part of point-of-care decision, we can conclude that it is not relevant to base one’s decision on the results of this RDT.

We hope you will find our revised version acceptable for publication in *PLOS Neglected Tropical Diseases* and look forward to hearing from you.

Yours sincerely,

Dr Olivier MAILLARD, PharmD, MSPH

Dr Antoine BERTOLOTTI, MD, PhD
