# Supplementary material for: Early diagnosis of dengue: Diagnostic utility of the SD BIOLINE Dengue Duo rapid test in Reunion Island
Source: PLoS Negl Trop Dis. 2023 Mar 30;17(3):e0011253. doi: 10.1371/journal.pntd.0011253 (PMC10089357; doi:10.1371/journal.pntd.0011253)
Supplement: Attachment — Submitted filename: Reviewer Comments.docx [file pntd.0011253.s006.docx]

**Reviewer’s Comments**

1. This type of study for evaluation of usefulness of RDTs for rapid diagnosis of Dengue has been published so many times in various journals previously. This is not a new/recent finding published in the present study.
2. the RT-PCR test carried out in this study was Tropical Fever Core multiplex RT-PCR (Fast Track Diagnostics®). This is a Multiplex RT-PCR having multiple targets apart from Dengue.
   a. It is not understood as to why a Multiplex RT-PCR kit was used while studying a single virus i.e. Dengue.
3. It is not clear as to how was the samples size calculated for arriving to this conclusion?
